# The human microbiome and juvenile idiopathic arthritis

**DOI:** 10.1186/s12969-016-0114-4

**Published:** 2016-09-20

**Authors:** Anouk Verwoerd, Nienke M. Ter Haar, Sytze de Roock, Sebastiaan J. Vastert, Debby Bogaert

**Affiliations:** 1 Laboratory of Translational Immunology, University Medical Centre Utrecht, Heidelberglaan 100, 3584 CX Utrecht, The Netherlands; 2 Department of Paediatric Rheumatology, Wilhelmina Children’s Hospital, Lundlaan 6, 3584 EA Utrecht, The Netherlands; 3 Department of Paediatric Infectious Diseases, Wilhelmina Children’s Hospital, Lundlaan 6, 3584 EA Utrecht, The Netherlands

**Keywords:** Juvenile idiopathic arthritis, Microbiome, Microbiota, Dysbiosis, Autoimmunity

## Abstract

Juvenile idiopathic arthritis (JIA) is the most common rheumatic disease in childhood. The pathogenesis of JIA is thought to be the result of a combination of host genetic and environmental triggers. However, the precise factors that determine one’s susceptibility to JIA remain to be unravelled. The microbiome has received increasing attention as a potential contributing factor to the development of a wide array of immune-mediated diseases, including inflammatory bowel disease, type 1 diabetes and rheumatoid arthritis. Also in JIA, there is accumulating evidence that the composition of the microbiome is different from healthy individuals. A growing body of evidence indeed suggests that, among others, the microbiome may influence the development of the immune system, the integrity of the intestinal mucosal barrier, and the differentiation of T cell subsets. In turn, this might lead to dysregulation of the immune system, thereby possibly playing a role in the development of JIA. The potential to manipulate the microbiome, for example by faecal microbial transplantation, might then offer perspectives for future therapeutic interventions. Before we can think of such interventions, we need to first obtain a deeper understanding of the cause and effect relationship between JIA and the microbiome. In this review, we discuss the existing evidence for the involvement of the microbiome in JIA pathogenesis and explore the potential mechanisms through which the microbiome may influence the development of autoimmunity in general and JIA specifically.

## Background

Juvenile idiopathic arthritis (JIA) is the most common rheumatic disease in childhood, with a prevalence of 16−150 cases per 100,000 children [1]. By definition, it is characterised by arthritis of unknown origin persisting for more than 6 weeks, and starting before the age of 16 years [1, 2]. Several different types of JIA can be defined, among which are oligoarticular, polyarticular and systemic JIA. This review will focus on oligoarticular and polyarticular JIA, for which an underlying autoimmune pathogenesis is assumed [1]. Despite recent advancements in our understanding of the inflammatory process that characterises JIA and the development of new treatment strategies, the precise factors that determine one’s susceptibility to JIA remain to be identified.

The microbiota has recently received increasing attention as a potential contributing factor to the development of a wide variety of health problems, including immune-mediated diseases [3]. The microbiota is a collective term for the trillions of microorganisms that inhabit our epithelial surfaces, including the gut, respiratory tract and skin. The collection of genes encompassed by the microbiota is referred to as the “microbiome”. Recent advances in high-throughput sequencing have enabled the detailed study of the composition of the microbiome and host-microbe interactions [4]. These studies have revealed that the complex interplay between the immune system and the microbiota is essential for a healthy development of the immune system and for protecting the host against colonization, overgrowth and invasion of pathogens [3]. The disruption of this mutualistic relationship can lead to an imbalance in the composition of the microbiota, or “dysbiosis”, which is thought to play a role in the development of numerous immune-mediated diseases, such as inflammatory bowel disease, type 1 diabetes, and rheumatoid arthritis (RA) [5–7].

Factors that have been associated with dysbiosis so far are diet and lifestyle, pathogens and drug use – especially the use of antibiotics [8–10]. Indeed, preliminary studies have demonstrated that the composition of the microbiome may be altered in JIA [11]. Furthermore, recent publications have suggested that early life antibiotic use is associated with a higher risk of development of JIA later in life [12, 13]. In this review, we explore the potential role of the microbiome in the pathogenesis of JIA, and provide an overview of the current evidence supporting this hypothesis.

### Disease mechanisms in JIA

The pathogenesis of JIA is currently thought to be the result of a combination of factors: in a genetically susceptible individual, exposure to one or more environmental triggers may lead to local tissue damage and/or the release of self-antigens, finally resulting in chronic synovial inflammation [1]. Preliminary evidence for a genetic component in the underlying aetiology of JIA initially came from twin and family studies. These studies have demonstrated a higher prevalence of JIA in twin and non-twin siblings of children with JIA, as well as similarities in disease course. Concordance rates between monozygotic twins were found to be 20–40 % [14, 15]. However, one must note that other non-genetic factors, including the composition of the microbiome, are highly similar in twins as well [16], making it difficult to differentiate between these factors. Recently, a large genome-wide association study with more than 2800 JIA cases and more than 13,000 healthy controls, however, indicated that only 18 % of JIA pathogenesis could be attributed to genetic factors [17]. The remaining part should therefore be explained by non-hereditary, possibly environmental influences these children commonly experience.

With respect to genetic susceptibility, multiple specific susceptibility genes have been identified, which can be broadly subdivided in HLA and non-HLA genes and differ between subtypes of JIA. For example, oligoarticular JIA is associated with HLA-A2, -DR5 and -DR8, whereas rheumatoid factor (RF-) negative polyarticular JIA is associated with the DRB1*08 and DBP1*03 haplotypes. RF-positive polyarticular JIA, on the contrary, is associated with DRB1*04, DQA1*03 and DQB1*03 [18]. The strong association of HLA-genes with JIA underscores the importance of T cells in pathogenesis of disease. Non-HLA genes that are linked to JIA mostly relate to the immune system and its cytokines. An example is *IL2RA*, encoding the alpha chain of the interleukin (IL)-2 receptor, which is associated with persistent oligoarticular JIA [2]. Other examples are macrophage inhibiting factor (MIF) and tumour necrosis factor (TNF), which are both associated with JIA in general [2]. One can imagine that these polymorphisms also indirectly influence the composition of the microbiome by having an effect on the immune response in general.

Although these genetic factors are important in explaining part of the complex pathogenesis of JIA, as said, concomitant environmental triggers may too likely play an important role. Infectious agents are considered as one of the potential environmental triggers of JIA pathogenesis [18, 19], possibly due to molecular mimicry of bacterial peptides with self-antigens. Both viruses (e.g. Parvovirus B19 and Epstein-Barr virus) and bacteria (e.g. Enterobacter spp., *Chlamydophila pneumoniae* and Streptococcus spp.) were studied as potential trigger of disease, but results remain inconclusive due to the unavailability of controlled prospective studies that are needed to identify drivers of disease in a time-dependent fashion.

On a cellular level, however, immune responses towards self-antigens are hypothesised to be a central event in the development of JIA. This is supported by the observation of clustering of memory T cells around antigen-presenting cells in the synovium of JIA patients, which are predominantly IFNγ^+^ and of the Th1 phenotype [1, 20]. Also T helper 17 (Th17) cells are, among others, found in the joints of children with JIA [21]. Interestingly, it was found that shifting from a Th17 phenotype to a Th17/Th1 or Th1 phenotype can occur in the synovial fluid of oligoarticular JIA patients during the disease course, underlining the involvement of different sets of effector T cells in the chronic synovial inflammation central to JIA [22]. In addition, regulatory T cells (Tregs) have been implicated in JIA and other autoimmune diseases because of their role in regulating and dampening aberrant immune responses [23]. In JIA, increased numbers of Tregs are found at the site of inflammation, which raises the question whether they retain their suppressive capacity at the site of inflammation. Indeed, effector T cells in the synovial fluid have shown to be resistant to suppression by Tregs [24]. Altogether, these observations contribute to a disease concept of JIA in which the balance between regulatory (T) cells and effector (T) cells is disturbed, resulting in chronic joint inflammation.

In addition to the adaptive immune system, it is now increasingly understood that also the innate immune system plays a role in the disease mechanisms of JIA [2, 25]. This is for example illustrated by the role of granulocyte-macrophage colony stimulating factor (GM-CSF), a potent inflammatory mediator responsible for the induction of innate immune cells, such as neutrophils and monocytes, in autoimmune disease. Interestingly, a recent study postulated that Th17 plasticity is a key driver of GM-CSF production in JIA tying these two observations together in one possible mechanistic route [26].

### The microbiome and the immune system

Encoding 9.8 million non-redundant genes and 100-fold more proteins than the human genome [27, 28], the gut microbiome provides an enormous source of antigenic variation. This variation is indispensable for a healthy development of the immune system, and helps the immune system recognise the normal microenvironment. The reciprocal interaction between the host and microbiota offers mutual benefits: while the host provides the microbiota with a niche and essential nutrients, the microbiota regulates a variety of physiological processes essential to the host [3]. Among others, the microbiotacontributes to the production of vitamins, and prevents invasion of pathogens by competing for nutrients [3, 29]. Furthermore, the host’s immune response to the microbiotamay also impact and shape the microbial ecosystem in the long term [3, 30]. On the other hand, the microbiota might potentially play a role in the generation of autoimmunity by triggering the immune system in an aberrant way or inducing cross-reactivity to self-antigens [8]. The balance between tolerance and activation seems delicate again: the immune system must be able to respond to any potentially harmful non-self antigen, while it should not eliminate its indispensable commensals [8].

#### The microbiome influences development of the immune system

The microbiome shapes the host immune system in a variety of ways. First, it affects development of the intestinal mucosal barrier itself [3, 31]. Underdevelopment of this barrier, possibly through an imbalance in the microbial community and release of inflammatory triggers like LPS, might lead to increased gut permeability, which in turn might result in the spread of bacterial components and (systemic) inflammation [31]. Secondly, studies using germ-free mice have demonstrated that the microbiome is essential for the normal generation and maturation of gut-associated lymphoid tissue (GALT) [32, 33]. Since GALT are structures in which antigenic presentation takes place, normal development of GALT is important for a healthy development of the immune system.

In addition to GALT, the microbiome was found to have a direct influence on the development of T cells, among others Th1 and Th17 cells [34, 35]. Th17 cells preferentially develop in the intestinal mucosa [36]. Numbers of Th17 cells were observed to be significantly reduced in antibiotic-treated and germ-free mice, implicating a role for the microbiome in Th17 immune development [34]. Restoring the microbiome of germ-free mice with stools of healthy mice results in normalization of intestinal Th17 cell numbers. Although the precise mechanism by which the microbiome induces Th17 development remains to be elucidated, it was found in one study that murine segmented filamentous bacteria (SFB), which are non-culturable *Clostridium*-species, seem to induce Th17 cell development in the lamina propria of the small intestine [35]. Importantly, Th17 cells can transform into Th1 cells under the influence of IL-22 or IL-23 signalling [37], as was described above for JIA [22].

In a similar fashion, the microbiome composition appears to influence the development of Tregs. Levels of Tregs were significantly reduced in germ-free mice [38]. Studies have shown that specific populations of commensal bacteria may induce the development of Tregs in mice, including again *Clostridium* spp., especially Clostridia clusters IV, XIVa and XVIII [38]. This was found to occur through the production of short-chain fatty acids (SCFAs), especially butyrate, which inhibit histone deacetylases. As histone deacetylases normally result in Treg induction, inhibition by butyrate would decrease Treg numbers. [39]. Also colonization of *B. fragilis*, a human commensal, was found to induce Tregs in mice [3].

As outlined above, Th1 cells, Th17 cells and Tregs are generally assumed to play a central role in development of autoimmunity [40]. Imbalance between these cell subsets resulting from the possible induction of Th1/Th17 cells over Tregs might trigger or at least sustain autoimmune responses. A recent review by Rogier et al. poses that the imbalance might be triggered by the microbiome community members through activations of Toll-like receptors (TLRs) [41]. TLR activation on antigen-presenting cells (APCs) enhances the antigenic signal to T cells by inducing the upregulation of MHCII, co-stimulatory molecules and the release of cytokines, and thereby shapes T helper cell subsets [41].

#### The microbiome as mediator of autoimmunity?

As described above, accumulating evidence suggests that the microbiome influences the development and function of the immune system. Furthermore, the cellular mechanisms behind these interactions are beginning to be unfolded. However, the question remains how microbes can act as mediators/inducers of autoimmune responses and contribute to autoimmune disease. A number of theories have been developed, of which molecular mimicry is the most well-known.

Molecular mimicry refers to the phenomenon in which a foreign antigen has structural similarities with self-antigens, typically leading to the formation of cross-reactive antibodies and T cells [8, 42]. Rheumatic fever is a well-known example in which severe systemic disease is caused by the generation of cross-reactive antibodies against group A streptococcus [43]. In addition, accumulating evidence suggests that commensals can induce cross-reactive lymphocytes [44, 45]. However, under physiological conditions, cross-reactive T cells should be more of the regulatory phenotype [8]. This therefore raises the hypothesis that as a result of immune-modulatory effects of the microbial community as described above, skewing of this cross-reactive immune response towards a more pathogenic T cell subset might explain the autoimmune phenomena. In addition, skewing of T cell responses may also occur in cases of mucosal barrier disruption. Studies have shown that a break in T cell tolerance may occur as a result of disruption of the intestinal mucosal barrier following invasion of pathogens [8, 46]. Since the microbial community in the intestine is highly involved in mucosal integrity and barrier function, both processes of immune-skewing and breakage of T cell tolerance might occur in parallel as a consequence of a physiological response, however, eventually leading to autoimmune inflammation.

Another possible mechanisms by which the microbiome may contribute to the generation of autoimmunity is bystander activation, which might occur with or without epitope spreading [47, 48]. Bystander activation refers to an indirect activation of autoreactive cells caused by the release of pro-inflammatory cytokines during inflammation or tissue damage, for example as a result of a dysbiotic environment. Epitope spreading occurs when an immune response directed against a specific (microbial) antigen includes different portions of the same or similar proteins [49, 50]. This activates a broader set of T cells, which in physiological circumstances helps to support the efficient elimination of the pathogen. However, under unbeneficial conditions, this might also lead to the indirect activation of autoreactive T cells thereby contributing to the generation of autoimmunity [8, 47].

Lastly, some microbes might alter host proteins, thereby creating new antigens that are recognized by the adaptive immune system as non-self [47]. An example is *Porphyromonas gingivalis,* which is able to citrullinate host proteins, thereby inducing the characteristic antibodies to citrullinated protein antigens (ACPAs) in RA [51].

### Evidence for involvement of the microbiome in JIA

Considering the delicate interaction between the immune system and the microbiome, and the recent evidence suggesting that the human microbiome may play a role in the generation of autoimmunity in general, one can imagine that the interaction of the immune system and the microbiome may contribute to the development of JIA. Multiple studies have addressed the potential role of the microbiome in JIA, which are summarised in Table 1. In addition to these studies, which predominantly included patients with oligo- and polyarticular JIA, the role of the microbiome in the pathogenesis of enthesitis-related arthritis (ERA) – another distinct subtype of JIA – was also studied and already reviewed comprehensively by Gill et al. (section on juvenile spondyloarthritis) [52, 53]. For example, Malin et al. studied bacterial enzyme activities in faeces of JIA patients and compared this to healthy controls [54]. They found increased bacterial urease activity in faecal samples of JIA patients, and reasoned that this might be a result of alterations in the anaerobic bacterial flora [54]. They also showed that by the administration of a human *Lactobacillus* strain, faecal urease activity could be again diminished [55]. In addition, Picco et al. reported increased intestinal permeability in all subtypes of JIA, further suggesting aberrations in mucosal homeostasis in JIA, although data on microbiome composition were lacking at this stage [56]. New cross-sectional studies investigating the composition of the microbiome in JIA patients indeed show that the gut microbiome is disturbed during disease. A pilot study among DMARD-naïve polyarticular JIA patients revealed that intestinal microbiome diversity within the phylum Firmicutes is reduced [57]. Furthermore, a recent study conducted by Tejesvi et al. analysed the composition of the faecal microbiota of newly diagnosed, immunosuppressant-naive oligo- and polyarticular JIA patients [11]. At the phylum level, they found a higher abundance of Bacteroidetes in JIA patients, whereas the abundance of Firmicutes was reduced. Likewise, at the genus level, a higher abundance of *Bacteroides* was found in children with JIA compared to controls [11]. Both of these findings are in accordance with what has previously been described in type 1 diabetes [6].

**Table 1:** Evidence for the involvement of the microbiome in JIA

Author & year	Patients & healthy controls (n)	Medication use	Study conclusion
Aberrations in mucosal homeostasis			
Malin 1996 [54]	Oligo-JIA (19) Poly-JIA (6) ERA (1) HC (26)	Mixed	↑ Bacterial urease activity in faeces of JIA patients
Picco 2000 [56]	Oligo-JIA (26) Mixed other (14)	Unknown	↑ Intestinal permeability in all subtypes of JIA
Microbiota composition			
Hissink-Müller 2013 [57]	Poly-JIA (8) HC (24)	DMARD naive	↓ Firmicutes in JIA patients
Tejesvi 2016 [11]	Oligo-JIA (13) Poly-JIA (16) ERA (1) HC (27)	DMARD naive	↓ Bacteroidetes;↓ Firmicutes in JIA Genera *Actinobacteria* and *Fusobacteria* only present in JIA
Childhood antibiotic use			
Horton 2015 [13]	JIA (152) HC (1520)	Unknown	Association between antibiotic exposure and JIA (dose and time-dependent); No association with nonbacterial antimicrobial agents
Arvonen 2015 [12]	JIA (1298) HC (5179)	Unknown	Association between antibiotic exposure and JIA (dose-dependent); strongest for lincosamides and cephalosporins

*Abbreviations*: *HC* healthy control, *ERA* enthesitis-related arthritis; ↑ = increased, ↓ = decreased

These cross-sectional studies intriguingly demonstrate that microbial communities are disrupted during disease. It can be assumed, however, that the disease itself also induces changes in microbial composition due to associated long-term (systemic) inflammation, catabolism, or altered behaviour induced by the symptoms of JIA. The question thus remains as to whether the observed microbial dysbiosis is cause or effect of the disease, or even a bystander effect, since these studies still lack an associative character with disease pathogenesis.

Recently, two case-control studies exploring the association between antibiotic exposure and the consecutive development of JIA, did attempt to unravel cause and effect relationships [12, 13]. These data are particularly interesting since it involves the use of antibiotics in childhood, in which the microbiota is still developing and therefore more vulnerable to disruption [58]. Arvonen et al. confirmed the association between exposure to antibiotics and risk of JIA, and additionally, demonstrated that the risk increased with repeated exposure [12]. They observed the strongest associations with treatment with lincosamides (e.g. clindamycin) and cephalosporins, which especially target gram-positive microorganisms leaving gram-negative (LPS-producing) bacteria unharmed. Horton et al. reported a similar association between antibiotic exposure and JIA development in a retrospective case control study analysing data from >500 general practitioners in the UK. They confirm an increase in risk with cumulative antibiotic exposure [13]. In addition, they observed the strongest association with antibiotics prescribed within 1 year preceding the diagnosis: interestingly in contrast to antibacterial drugs, non-bacterial antimicrobial agents (i.e. antiviral or antifungal) were not associated with the development of JIA [13]. A growing body of evidence suggests that even short-term exposure to antimicrobial agents may have lasting effects on the quantity and composition of the intestinal microbiota in general [10].

The major limitation of these case-control studies is the risk of confounding: the association between antibiotic use and the development of JIA may also reflect, for example, treatment of the first symptoms of JIA with antibiotics, or alternatively be attributed to increased susceptibility to infections in JIA [12]. Infections themselves may even explain both the increased use of antibiotics, microbiota dysregulation and JIA susceptibility. In addition, conflicting data arise from these case-control studies, cross-sectional studies (e.g. Tejesvi et al.), and fundamental research. For example, clindamycin – one of the antibiotics which use has been associated with JIA, as described above [12] – specifically targets anaerobic bacteria, and has indeed been shown to have a negative impact on the intestinal microbiota [59, 60]. However, *Bacteroides,* an important group of these anaerobic bacteria dominating the gut microbiota, has been implicated to be essential for the maintenance of functional stability of the human gut [10]. In addition *Bacteroides, s*pecifically B. fragilis, is regarded to be critical for adequate Treg development [61]. Contrastingly, Tejesvi et al. describe an increase in the phylum Bacteroidetes as opposed to a decrease what would be expected if clindamycin played a major role [11]. One explanation for the contradictory evidence may be that it is not the increase or decrease in Bacteroidetes that matters most, but rather the ratio between Bacteroidetes and Firmicutes or changes in specific species within a population, e.g. Clostridia of the phylum Firmicutes, which was also found to be reduced [11, 57]. Following clindamycin treatment, an increase in Bacteroidetes could also be explained by the generation of clindamycin-resistant species, which has previously been described [60, 62].

From the observations made so far, one may at least conclude that there is evidence for microbial dysbiosis in JIA patients. However, the question as to what triggers this shift, what bacteria are actually beneficial or harmful, and whether the associations are part of cause and effect relationships, remains unanswered. Longitudinal studies, preferably cohort studies, should be performed in order to investigate whether the microbiome has a causal relationship to the development of JIA.

Another interesting thought is whether diet can explain part of the link between microbiota dysregulation and JIA, especially considering the differences in JIA epidemiology throughout the world. It is known that the overall prevalence of JIA is much lower in Asia than in Europe and North America [2]. Also the prevalence of JIA subtypes varies widely, where oligoarticular JIA is the most common subtype among Caucasians but much less frequently occurs in, for example India [63]. It is known that diet influences the composition of the microbiota across geographic areas [64]. One must, however, also take into account that genetics and environment also strongly differ throughout the world, which renders it difficult to draw definite conclusions on the role of diet alone.

### Future challenges and perspectives

**Fig. 1:**
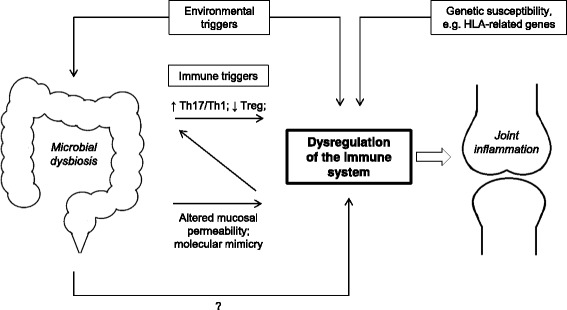
*The potential role of the microbiome in the pathogenesis of JIA*. Microbial dysbiosis may result in dysregulation of the immune system through influencing the interaction with T cell subsets and the integrity of the mucosal barrier. Altered mucosal permeability may in turn also influence the direction in which T cell subsets develop. Together with genetic susceptibility and environmental triggers, this may result in autoimmunity and joint inflammation

Taking into consideration both previous thoughts and new insights, we propose an updated model for the pathogenesis of JIA that integrates microbial dysbiosis (Fig. 1). However, much work still needs to be done in order to verify the associations that are made and unravel the mechanistic underpinnings.

A number of important questions remain outstanding. For instance, it is likely that next to environmental factors, host genetics may also impact the composition of our microbiome, and thereby play a crucial early role in defining disease susceptibility. In contrast, one might also reason that dysbiosis is primarily the result of external environmental factors, and that genetic factors only come to play a role when dysbiosis is already present. Another interesting question is whether disease results from small or rather broad alterations in microbial communities, and whether either the absence or the presence of specific bacteria are key. Furthermore, next to the gut microbiome, there are now indications that also the microbiome from other body sites, such as the oral cavity and respiratory tract, may play a role, which was recently reviewed extensively for RA [65] – although these might likely be co-associated with alterations in gut microbiota, since all these niches are communicating and therefore can be all considered part of the human microbiome.

A deeper understanding of the pathways by which disturbances in the microbiome may evolve to disease may open doors to the development of new treatment or prevention strategies in the future. One approach to restore the normal healthy gut microbiome is to replace it by means of Faecal Microbiota Transplantation (FMT), which proved to be very effective in the treatment of refractory *Clostridium difficile* infections as well as in ulcerative colitis [66, 67]. Alternatively, an attempt could be made to restore the microbial community by introducing anti-inflammatory commensals through pro-/prebiotics or dietary interventions. Interestingly, initiatives are now being taken to culture targeted combinations of bacteria in vitro that can restore intestinal homeostasis [68, 69].

## Conclusions

A growing body of evidence indicates involvement of the gut microbiome in the pathogenesis of JIA. Nevertheless, much work still needs to be done to understand how the interplay between genetics, the microbiome and other possible environmental factors eventually results in the development of chronic immune-mediated arthritis, such as JIA. A better understanding of the interactions between the host and microbiome may help to establish alternative ways in which to treat, or even prevent disease.

## Abbreviations

ACPA: Antibodies to citrullinated protein antigens
APC: Antigen-presenting cell
DMARD: Disease-modifying anti-rheumatic drug
ERA: Enthesitis-related arthritis
FMT: Faecal microbiota transplantation
GALT: Gut-associated lymphoid tissue
GM-CSF: Granulocyte macrophage colony-stimulating factor
HLA: Human leukocyte antigen
IL: Interleukin
JIA: Juvenile idiopathic arthritis
MIF: Marcophage inhibiting factor
OTU: Operational taxonomic unit
RA: Rheumatoid arthritis
SCFA: Short-chain fatty acids
Th17: T-helper 17
TLR: Toll-like receptor
TNF: Tumour necrosis factor
Treg: Regulatory T cell
